# Supplementary material for: Potential of Transcript Editing Across Mitogenomes of Early Land Plants Shows Novel and Familiar Trends
Source: Int J Mol Sci. 2019 Jun 18;20(12):2963. doi: 10.3390/ijms20122963 (PMC6627324; doi:10.3390/ijms20122963)
Supplement: Supplementary file 1 [file ijms-20-02963-s001.zip › Supplementary_Figures.pdf]

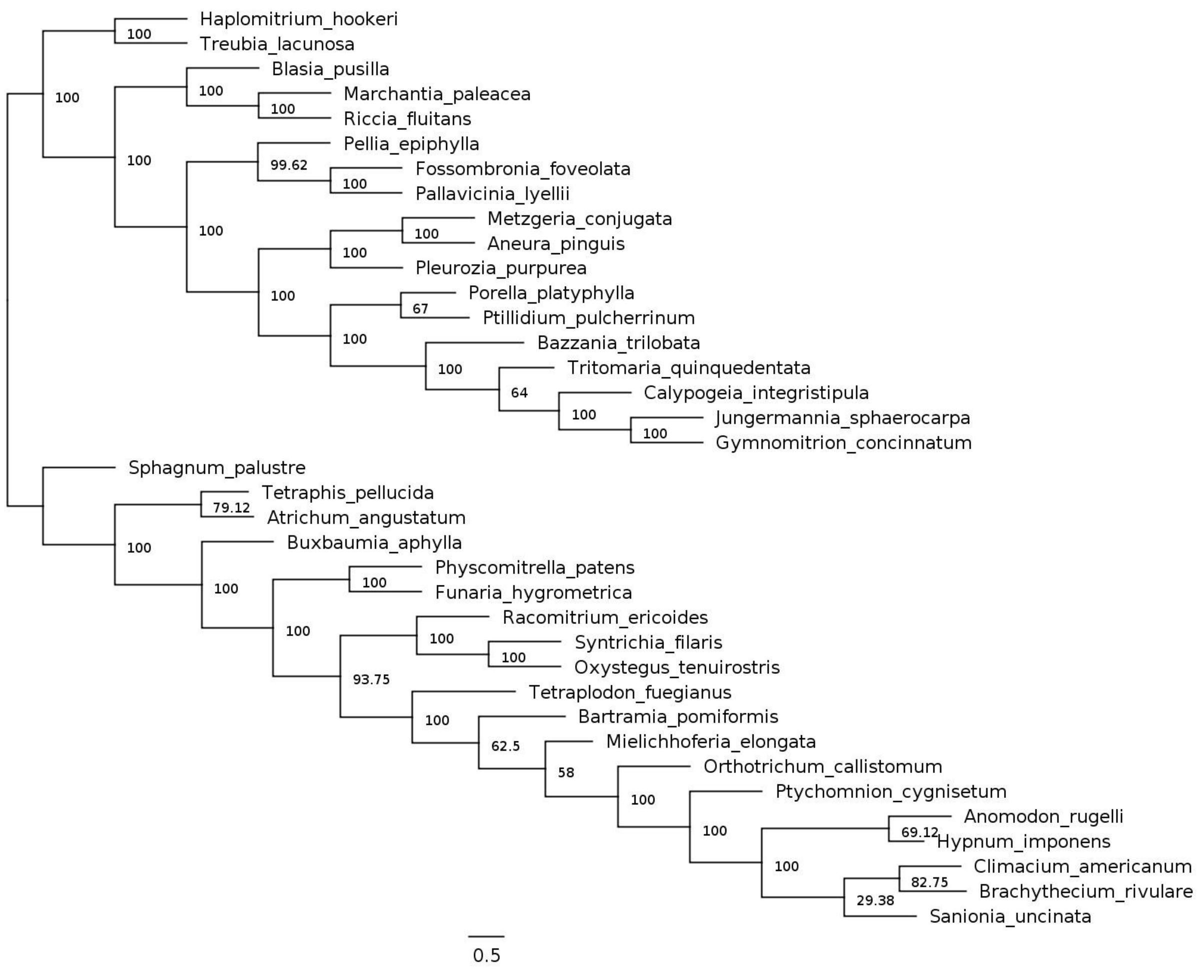

Figure S1. Phylogenetic relationships of 37 species of liverworts and mosses based on 33 concatenated mitochondrial protein-coding sequences. The phylogenetic tree was obtained as a result of Maximum Likelihood analysis. The bootstrap values are shown at the nodes. The scale bar indicates the number of substitutions per nucleotide position.

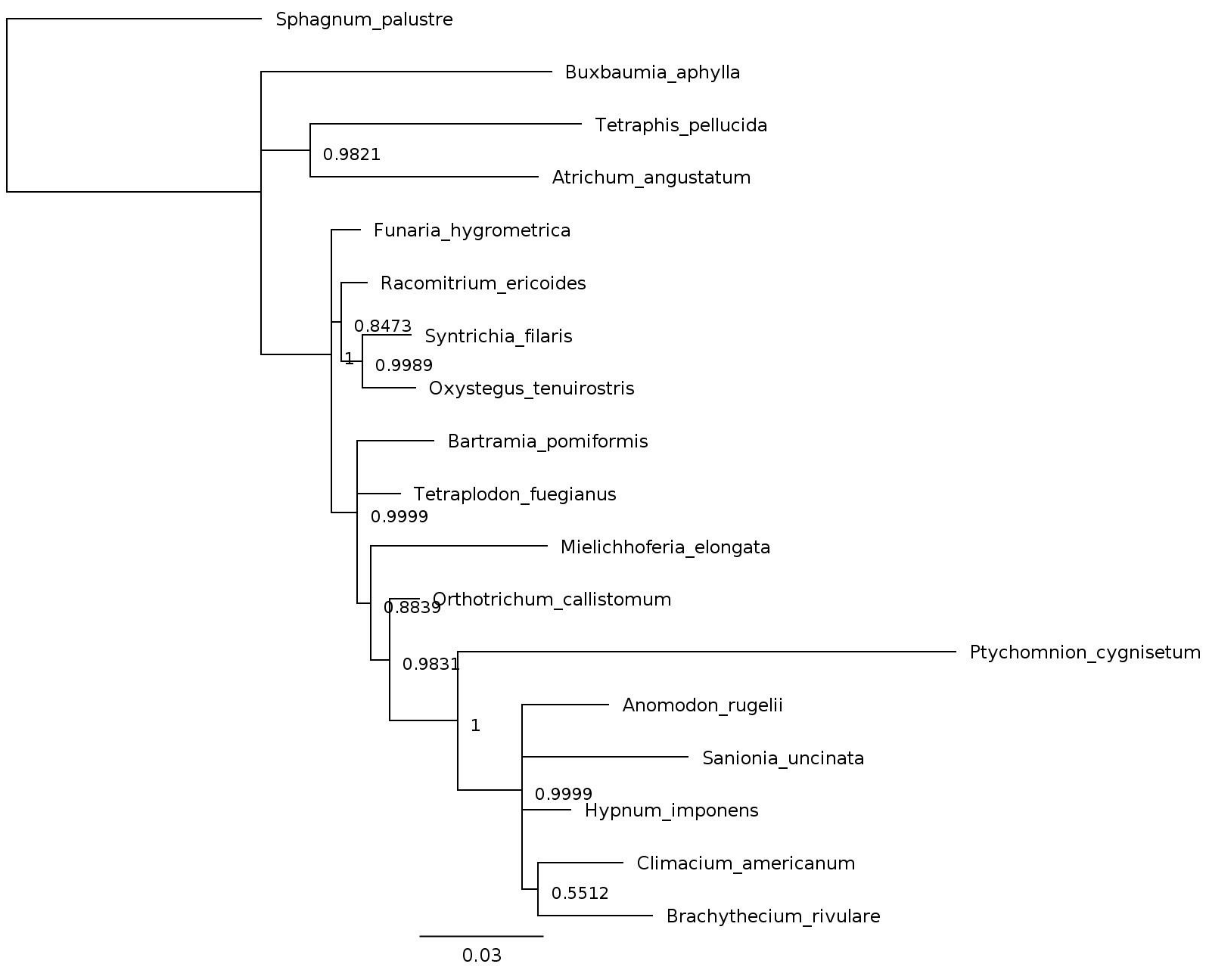

Figure S2. Phylogenetic relationships of 18 species mosses based on binary-state matrix of predicted C-to-U editing sites positions. The phylogenetic tree was obtained as a result of Bayesian analysis. The posterior probability values are shown at the nodes. The scale bar indicates the number of substitutions per nucleotide position.

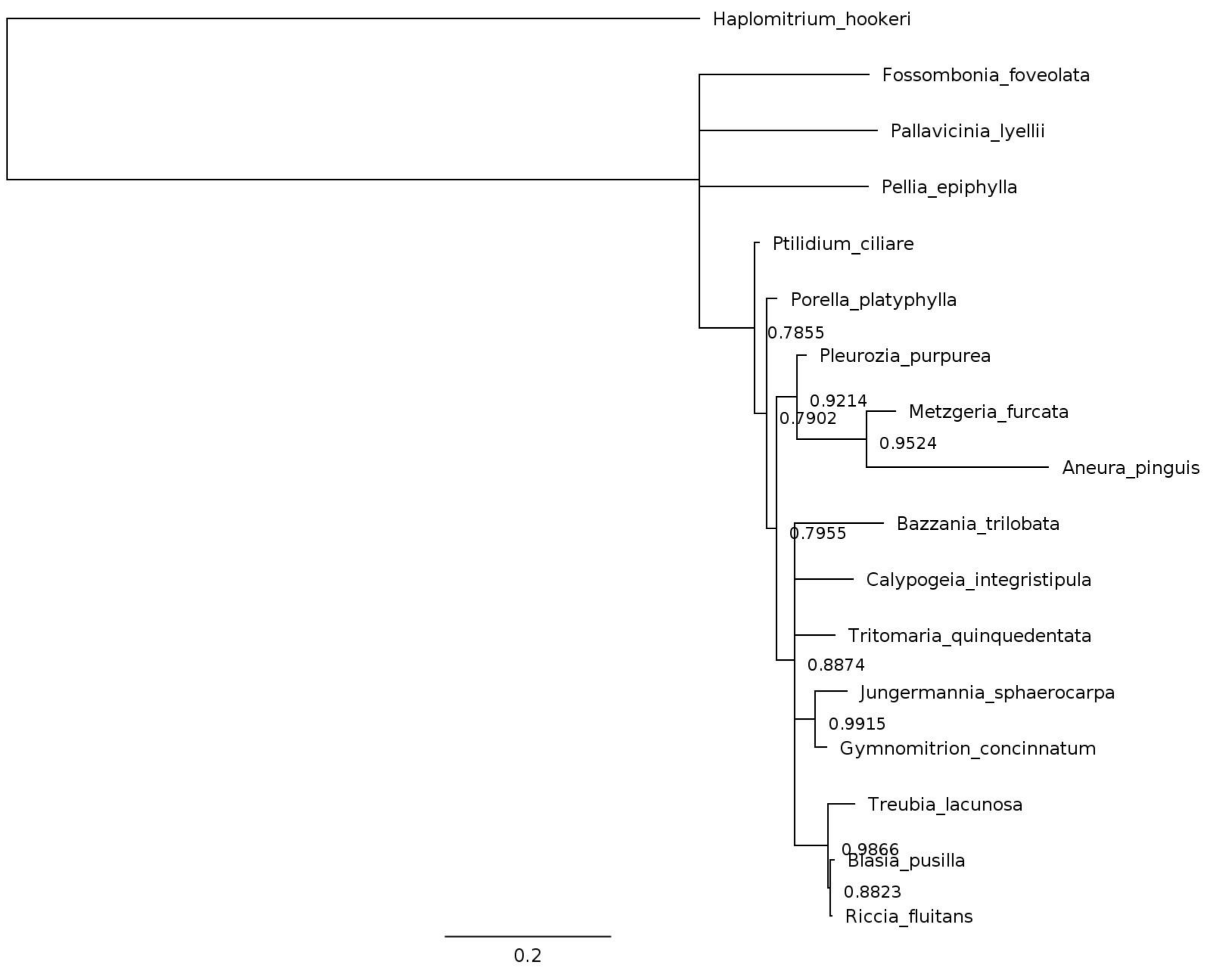

Figure S3. Phylogenetic relationships of 17 species liverworts based on binary-state matrix of predicted C-to-U editing sites positions. The phylogenetic tree was obtained as a result of Bayesian analysis. The posterior probability values are shown at the nodes. The scale bar indicates the number of substitutions per nucleotide position.
